# “*I just believe there is a risk*” understanding of undetectable equals untransmissible (U = U) among health providers and HIV‐negative partners in serodiscordant relationships in Kenya

**DOI:** 10.1002/jia2.25466

**Published:** 2020-03-06

**Authors:** Kenneth Ngure, Fernandos Ongolly, Annabell Dolla, Merceline Awour, Kenneth K Mugwanya, Elizabeth Irungu, Nelly Mugo, Elizabeth A Bukusi, Jennifer Morton, Josephine Odoyo, Elizabeth Wamoni, Gena Barnabee, Kathryn Peebles, Gabrielle O'Malley, Jared M Baeten, for the Partners Scale‐Up Project Team

**Affiliations:** ^1^ Department of Community Health Jomo Kenyatta University of Agriculture and Technology Nairobi Kenya; ^2^ Department of Global Health University of Washington Seattle WA USA; ^3^ Center for Clinical Research Kenya Medical Research Institute Nairobi Kenya; ^4^ Center for Microbiology Research Kenya Medical Research Institute Nairobi Kenya; ^5^ Department of Epidemiology University of Washington Seattle WA USA; ^6^ Department of Medicine University of Washington Seattle WA USA

**Keywords:** HIV viral suppression, treatment as prevention, ART, PrEP, HIV serodiscordant couples, risk, Kenya

## Abstract

**Introduction:**

Sustained HIV viral suppression resulting from antiretroviral therapy (ART) eliminates the risk of HIV transmission, a concept popularly framed as Undetectable = Untransmittable (U = U). We explored knowledge and acceptance of information around the elimination of HIV transmission risk with ART (U = U) in Kenya.

**Methods:**

Our qualitative study was conducted within a project evaluating the use of pre‐exposure prophylaxis (PrEP) integrated into ART care for HIV serodiscordant couples in public clinics in Kenya (the Partners Scale Up Project). From February 2017 to April 2019, we conducted semi‐structured key informant interviews with 83 health providers and in‐depth interviews with 61 HIV‐negative people in serodiscordant relationships receiving PrEP services. Transcripts were coded using thematic analysis.

**Results:**

Health providers reported being aware of reduced risk of HIV transmission as a result of consistent ART use and used words such as “very low,” “minimal” and “like zero” to describe HIV transmission risk after viral suppression. Providers reported finding viral load results helpful when counselling clients about the risk of HIV transmission. Many lacked confidence in U = U and counselled on consistent condom use even after viral suppression while some expressed concerns that communicating this message to people living with HIV (PLHIV) would lead them to engage in multiple sexual relationships. Other providers reported that they did not counsel about the reduced risk of HIV transmission after viral suppression for fear of being blamed if HIV transmission occurred. HIV‐negative partners reported being informed about U = U by providers but they did not believe nor trust the message. Even after their partners achieved viral suppression, some HIV‐negative partners were unwilling to stop PrEP, while others indicated that they would use condoms if they stopped PrEP to be sure that they were protected from HIV.

**Conclusions:**

Despite awareness that effective ART use eliminates HIV transmission risk, there is both a lack of in‐depth knowledge and conviction about the strategy among health providers and HIV‐negative partners in serodiscordant relationships. New strategies that go beyond communicating the science of U = U to consider the local social and clinical environments could maximize the effectiveness of U = U.

## 1 Introduction

Studies conducted in diverse settings have demonstrated that effective use of antiretroviral therapy (ART) that results in viral suppression eliminates the risk of HIV transmission 1, 2, 3, 4. Global consensus supports that persons who have achieved and maintain undetectable viral load cannot transmit HIV sexually to their partners, and a popular campaign dubbed U = U (“undetectable [viral load] equals untransmittable”) has broadcast this message globally 5.

While studies provide evidence that ART can prevent HIV prevention, health providers in developed countries have reported challenges in implementing treatment as prevention as a public health strategy 6, 7, 8, 9. This has resulted in a disconnect between what providers know and what they communicate to people living with HIV (PLHIV) and their HIV‐negative partners. Studies conducted with gay and bisexual men have found low awareness of treatment as prevention 10, 11, 12. Additionally, HIV‐negative people report reluctance about engaging in condomless sex with HIV‐positive partners even after viral suppression unless they are on pre‐exposure prophylaxis (PrEP) 12, 13. Notably, there is a paucity of data from heterosexual couples and their providers especially in developing countries about their understanding of the U = U concept 14.

Kenya has the fourth largest HIV epidemic in the world 15. Both Kenyan 16 and WHO HIV treatment guidelines 17 recommend ART for all PLHIV. Kenya recommends PrEP for people with substantial on‐going HIV risk and is available free of charge in government facilities 16, 18. Among HIV serodiscordant couples, PrEP is recommended in Kenyan guidelines as a bridge to viral suppression, that is, until the HIV‐positive partner has been receiving ART for six months and ideally has been documented to have suppressed virus in plasma 19. This strategy of time‐limited PrEP use for couples requires health providers and PrEP users to be convinced that viral suppression eliminates HIV transmission (U = U). Therefore, research with HIV‐negative partners and their health providers offers an opportunity to investigate how people understand U = U and the consequences that understanding has on PrEP use patterns. We used qualitative interviews to explore the understanding of viral suppression and the resultant elimination of HIV transmission risk among health providers and PrEP‐using male and female heterosexual partners.

## 2 Methods

### 2.1 Study design and population

From February 2017 to April 2019, we conducted individual interviews with HIV‐negative women and men who were members of heterosexual HIV serodiscordant couples and health providers as part of the Partners Scale‐Up Project. The Partners Scale‐Up Project is evaluating PrEP delivery integrated into 30 public HIV clinics in Central (twelve clinics), Western (twelve clinics) and Coastal Kenya (six clinics) 20. In Kenya, PrEP is recommended for HIV‐negative partners until HIV‐positive partners initiate and sustain ART use for at least six months 18. Health providers in the 30 clinics were trained using a two‐day interactive curriculum on PrEP delivery and antiretroviral‐based HIV prevention 21. This training was aimed at catalysing PrEP delivery and specifically for serodiscordant couples the training emphasized that the HIV‐negative partner needed to use PrEP until the HIV‐positive achieved viral suppression, in agreement with Kenyan guidelines. U = U was included specifically as part of that training and was tested in pre‐ and post‐training evaluations. Providers were not employed by the Project and delivered HIV and PrEP care as part of their routine duties. Thus, we did not directly observe how information about U = U was conveyed with patients, and our findings therefore might reflect real‐world care delivery. Health providers and HIV‐negative partners were purposefully selected to ensure that the 30 clinics were all represented.

### 2.2 Data collection

We conducted key informant interviews with health providers and in‐depth interviews with HIV‐negative partners in serodiscordant relationships receiving PrEP services. We used semi‐structured interview guides. The HIV‐negative participants’ guide explored the views and confidence in elimination of HIV transmission after HIV suppression. The health providers’ guide explored provider views about how well ART works as HIV prevention and the information these providers give clients about how well treatment as prevention works. We did not specifically ask about the U = U slogan but more about the message behind it which is that viral suppression eliminates HIV transmission (interview guides included as Data S1 and S2). Guides were piloted and translated into Kiswahili and Dholuo. Interviews were conducted by trained social scientists. Interviews for the HIV‐negative partners were conducted in Kiswahili, Dholuo, or English based on interviewee preference and later translated to English. Interviews of health providers were conducted in English.

### 2.3 Data analysis

We performed a thematic analysis to produce a description of key concepts and themes arising within and between the individual primary categories represented in the interview guides 22. An initial codebook was developed both deductively from the interview guide and inductively from the transcripts. Transcripts were imported into Dedoose (Scientific Software Development GmbH, Berlin, Germany) for analysis. Transcripts were coded independently by at least two social scientists. Disagreements were resolved through discussion until consensus. Investigators used an iterative process of reading transcripts, comparing and contrasting coding, and identifying convergent and divergent themes within and between transcripts.

### 2.4 Ethical considerations

The study was approved by the University of Washington and Kenya Medical Research Institute ethics review boards and participants provided written informed consent for in‐depth interviews.

## 3 Results

We conducted qualitative key informant interviews with 83 health providers (thirty‐two clinical officers, seventeen nurse counsellors, fourteen adherence counsellors, five social workers, five health records officers, three HIV testing counsellors, three peer educators and one pharmaceutical technologist). The mean age for health providers was 35 years (23 to 65) and they had on average 49 (3 to 204) months of experience. Forty‐seven health providers were female (56.6%) and 36 (43.4%) were male. Additionally, we conducted interviews with 61 HIV‐negative people (35 (57.4%) females and 26 (42.6%) males) in serodiscordant relationships who were receiving PrEP.

### 3.1 Awareness of prevention benefits of viral suppression

Most health providers reported being aware that, with good adherence, PLHIV can achieve viral suppression within six months and used words such as “very low,” “minimal,” “like zero” and “close to zero” to describe the risk of HIV transmission after viral suppression. However, very few used the words “no risk.” Those who had more awareness were mainly providers with a clinical background compared to other providers working in HIV clinics.

“Definitely, when someone is virally suppressed, the chances of the transmission of HIV is like zero and ART has really helped in reducing new cases of HIV…” (Female, Clinical Officer, Central Region)

However, despite high awareness of the prevention benefits of viral suppression, deeper understanding of the underlying science was lacking. For example, some providers believed that U = U worked, but only in the context of consistent condom use while some likened viral suppression to the window period when HIV transmission can occur without HIV rapid tests showing an individual as HIV positive. Some expressed a need for further training to answer their “many questions” regarding U = U. Health providers (no matter their understanding or background) also reported that many of their colleagues did not understand the concept well.

“What I tell them is that it takes six months and you can only be suppressed when you adhere, when you use condoms and follow‐up with your clinics regularly.” (Female, HIV Counsellor, Central Region)

“That has also been a challenge, just explaining it to them they still don't understand it very well let alone the patients even the health workers themselves I don't think they understand it.” (Male, Clinical Officer, Coast Region)

Health providers provided mixed responses regarding U = U based on their clinical experience. Those who had witnessed that those who had viral suppression did not transmit to their partners believed in U = U. Others reported doubting U = U because they had observed HIV transmissions in HIV serodiscordant couples when the HIV‐positive partner was reportedly on ART, especially prior to the introduction of PrEP.

“ART works, so okay I am not going to talk about the scientific bit of it but what I have seen you find that a couple comes they are tested for HIV and you find that one is positive and the other is negative, then the positive partner discloses that they have been on ART for years and they have been having unprotected sex with the negative partner for years and they are still negative. So, we have had many of such cases so that is proof enough.” (Male, Clinical Officer, Coast Region)

“No, that one I cannot say that because formally when the PrEP was not being issued to these clients, you may find that the percentage that were…take for example those who were discordant, the percentage of those who were turning positive then were a bit high and imagine their partners were taking ARVs.” (Male, Peer Educator, Western Region)

Although many HIV‐negative partners reported being informed about the prevention benefits of viral suppression by their health providers together with their HIV‐positive partners, there were several who said that they did not fully understand the concept.

“I told you about viral load and whatever…I don't understand them very well. You see it suppresses the HIV germs, you see if they are low and he does not have these other diseases, it is very hard for him to infect me.” (Female, HIV‐negative partner, Central Region)

“I am not sure about that but I heard them teach us today that if one adhered to ART then the person may achieve viral load suppression and that may protect the other person from getting HIV from the partner who is HIV positive… (but) there is still a possibility that one who is on ART could still infect the negative partner.” (Female, HIV‐negative partner, Western Region)

### 3.2 Counselling on U = U in the context of PrEP

Health providers who believed in U = U described routinely counselling HIV‐positive patients and their HIV‐negative partners that with good adherence to ART they would achieve viral suppression and reduce the risk of HIV transmission, which they believed became a motivator for high adherence. Providers explained that the concept of U = U was new to many clients and found it easier to counsel about U = U when couples attended the clinic together. Additionally, many providers concurred that viral load tests confirming viral suppression should be done first so that the clinical team could use the results when counselling clients, increasing confidence in both providers and clients, which could also enhance counselling about PrEP discontinuation. Others said that although they had confidence in U = U they experienced difficulties in convincing clients that U = U worked.

“After six months the new client on ARV will be tested for viral load and if they have undetectable viral load, we say they can stop using PrEP but if the viral load is detectable, we say there is need to continue until the positive partner suppresses.” (Female, Nursing Officer, Central Region)

“It also depends on the attitude of the client, there are clients who accept PrEP positively so for those clients that accepts that they just continue with the PrEP so long as the virus is suppressed but there are clients who this PrEP it gives them a burden. Taking the drug daily it is affecting them so once the viral load is suppressed or ART more than six months, they are comfortable to discontinue PrEP.” (Male, Peer Educator, Western Region)

Health providers who did not believe in U = U said that they did not discuss U = U because they feared that their clients could seroconvert and blame them for their HIV status. Some said that poor knowledge of U = U made them lack confidence in counselling clients about U = U.

“R: Because I feel that still they can infect the other partner even if they are low and undetectable, if they want to stop using PrEP then they must use condoms.

I: So, you don't believe that the negative partner can now stop using PrEP once the positive partner has achieved viral suppression?

R: I don't believe in that even if they are low and undetectable and there are many discordant couples here that their HIV partners are already low and undetectable, but we have never told them that they should stop taking PrEP.” (Female, Peer Educator, Coast Region)

Many HIV‐negative partners reported that although they had been informed about U = U by their providers they did not believe or trust the message which led to their reluctance to discontinue PrEP after viral suppression. Confidence levels were higher among those who had practiced unprotected sex and had remained negative even before starting PrEP. Even after HIV‐positive partners attained viral suppression, most HIV‐negative partners were unwilling to stop PrEP or said that they would re‐initiate PrEP during conception attempts, while others reported that they would use condoms if they stopped PrEP. Other reasons given for continuing PrEP and/or condom use despite knowing the prevention benefits of ART was to maximize prevention and *make sure that I am 100% protected*. Some HIV‐negative partners required assurance from health workers that if they stopped PrEP, they would not get infected by HIV.

“I was told like when they do for him the viral load test, like the last time that he was done for the test I was told that his virus was now dormant, and it is like they were now dead. And so, they told me that then even if we had sex then he could not infect me with HIV…I don't believe because I know that he can infect me regardless of his viral load. (Short laughter) …. I don't believe in that because I like talking about what I have experience with like now I am taking PrEP and I have had sex with him, and I never got HIV. So, I am now 100% sure that PrEP works…”(Female, HIV‐negative Partner, Coast Region)

### 3.3 Concerns associated with U = U

Health providers described concerns which made them hesitant to emphasize U = U. Although many health providers reportedly counselled their clients that when they were virally suppressed the chances of them infecting their partners was low, some were concerned about the possibility of a viral rebound. These providers had reported observing clients’ viral loads fluctuating even after adhering well to ART and were worried that this could be an opportunity for an infection to occur once the HIV‐negative partners discontinued PrEP use.

“As a health provider you might realize that today you are virally suppressed and then six months or one year down the line you are no longer suppressed. My thinking is that when one considers to be on PrEP, let them continue being on PrEP.” (Female, HIV Counsellor, Central Region)

“In the trainings that I have attended the issue of viral load are taken annually and at times things happen that make the clients have a high viral load. If you wait for a year before you take the viral load, this client would have already been infected, just to be sure and you are not using condoms, why not continue with PrEP even if your partner is virally suppressed.” (Male, Linkage Care Navigator Central Region)

Additional concerns were expressed by some health providers who reported avoiding counselling about U = U even after viral suppression for fear they would be blamed if HIV transmission occurred. Other providers said that they believed that communicating the U = U message would encourage HIV‐positive people to engage in multiple sexual relationships, so the health providers did not discuss U = U to HIV‐positive patients even after viral suppression.

“It is effective because it suppresses the virus and if the viral load is undetectable or below 1000 copies there are very minimal chances of infecting other people with HIV but sometimes, we do not tell them that their chances of infecting others is minimal because some will get loose.” (Female, HTS Counsellor, Central Region)

Although some HIV‐negative partners believed in the concept of U = U they did not trust that their partner would remain undetectable. Others said that once their HIV‐positive partners achieved viral suppression, they (HIV negative partners) would take the responsibility for ensuring that their HIV‐positive partners adhered to their medication as a condition for their discontinuation of PrEP. Some HIV‐negative partners wondered if they could restart PrEP when their partners with HIV did not adhere to ART.

“I would never stop taking PrEP because I am not sure of her viral suppression.” (Male, HIV‐negative partner, Western Region)

## 4 Discussion

Although most health providers and HIV‐negative partners in serodiscordant relationships in our study in Kenya reported being aware of the prevention benefits of consistent ART use, many lacked confidence in this HIV prevention strategy. A deep understanding was lacking among health providers, which translated to challenges (and reluctance) in discussing U = U with patients. Health providers with clinical backgrounds (nurses and clinical officers) appeared to have better awareness and more positive attitude towards U = U and could therefore play a key role in counselling PLHIV and their partners. In addition, many HIV‐negative partners were unwilling to stop PrEP even after their HIV‐positive partners achieved viral suppression. This may reflect either poor understanding or lack of trust in the U = U concept or unwillingness to hand over prevention responsibility to HIV‐positive partners.

Health providers used language suggestive of reduced risk but rarely had the confidence to say that HIV transmission risk was eliminated after viral suppression. Health providers play a key role in communicating new health interventions, including U = U and PrEP 23, 24. The U = U knowledge gap has been reported in a recent global survey of clinicians 23. Consistent with other studies, there is therefore a need to support health providers on the science behind U = U so that they can effectively communicate U = U in a believable way 23, 25.

Although a majority of HIV‐negative partners were aware of the concepts underlying U = U, there were some who had not heard the message, and many did not believe it. Our findings are comparable to a study conducted in Zambia and South Africa that reported that people were largely unfamiliar with the prevention benefits of HIV treatment 14 and with studies done among gay men who expressed reluctance for condomless sex even after viral suppression 12, 13. Awareness and acceptance of the additional HIV prevention benefit of ART needs to go beyond targeting HIV‐positive partners; including HIV‐negative partners in U = U discussions could provide motivation for HIV‐positive people to adhere to ART.

There were health providers and HIV‐negative partners who believed in the science of U = U but did not trust that HIV‐positive people would be able to adhere well enough to achieve and maintain viral suppression. It is noteworthy that HIV incidence has not been substantially lowered in some universal test and treat community trials, which could impact effectiveness of the U = U message in programmatic settings 24, 25, 26. Health providers who had observed viral load rebound in patients were more reluctant to counsel on U = U. Similarly, HIV‐negative partners expressed concern regarding their partners’ ability to maintain adherence, reflecting well‐documented challenges of maintaining ART adherence in Africa 27, 28, 29, 30, 31.

Some health providers and HIV‐negative partners expressed a desire for HIV viral load results to confirm viral suppression and support counselling about U = U. In Kenya and many sub‐Saharan Africa countries, national ART guidelines recommend HIV viral load testing every six months, however the results are not always returned in a timely fashion and often testing is not done at all. Therefore, the concern raised by providers is a critical barrier to U = U implementation and the health system should be strengthened to support provision of timely viral load results. Additionally, innovations such as point of care viral load monitoring being implemented in some settings could help in the rollout of U = U in resource constrained settings and increase provider and patient confidence in this important strategy 32, 33, 34.

A key concern reported by health providers about U = U was the fear that this message would encourage PLHIV to engage in more sex without condoms. Health provider concerns about risk compensation led them to withhold U = U information to HIV‐negative partners, which raises ethical concerns and could be viewed as medical paternalism 35. Health provider training on U = U should include information and that condomless sex is not risky for HIV transmission after viral suppression and ethical obligations of conveying new medical information to patients and allowing them to make informed choices.

Our study has limitations. First, we did not conduct observations of health providers’ interactions with HIV‐negative partners, which could have provided additional insights into awareness and knowledge of U = U. Second, our study was conducted in the context of a large PrEP implementation project in 30 clinics where health providers had been trained on U = U; other health providers may be less informed than the providers in this study. Third, we did not seek the views of HIV‐positive partners who have been shown to have better understanding and acceptability of U = U in some studies 11, 12, 36.

## 5 Conclusions

Despite awareness that consistent ART use can reduce the risk of HIV transmission, a deeper understanding of U = U was lacking, and some health providers and PrEP users were reluctant to rely on the U = U strategy. Timely viral load results could help boost this confidence in viral suppression. There is need for innovative strategies to improve the confidence of health providers in the concept of U = U which could in turn be conveyed to the PLHIV and their HIV‐negative partners.

## Conflict of interest

The authors declare that they have no competing interests.

## Authors’ contributions

KN, GO and JMB conceptualized and designed the study. MA, AD and FO acquired the data. MA, AD, FO and KN analysed the data. KN and JMB wrote the paper. KN, FO, AD, MA, KKM, EI, NM, EAB, JM, JO, EW, GB, KP, GO and JMB reviewed and edited the manuscript and approved the final version.

## Funding

The Partners Scale‐Up Project is funded by the National Institute of Mental Health of the US National Institutes of Health (grant R01 MH095507) and the Bill & Melinda Gates Foundation (grant OPP1056051).

## Supporting information


**Data S1.** Health provider interview guide.

**Data S2.** PrEP user interview guide.
