# Supplementary material for: “I just believe there is a risk” understanding of undetectable equals untransmissible (U = U) among health providers and HIV‐negative partners in serodiscordant relationships in Kenya
Source: J Int AIDS Soc. 2020 Mar 6;23(3):e25466. doi: 10.1002/jia2.25466 (PMC7060133; doi:10.1002/jia2.25466)
Supplement: Supplementary file 2 — Data S2. PrEP user interview guide. [file JIA2-23-e25466-s002.doc]

**An implementation project to scale-up delivery of antiretroviral-based HIV prevention**

**among Kenyan HIV serodiscordant couples (Individual interviews)**

Qualitative component: To gain a deeper understanding of HIV uninfected partner’s experiences with services with an aim to getting information that could be used to improve the services.

***Target group*: HIV uninfected persons enrolled in the Partners Scale-up Project**

*This interview guide is for the HIV uninfected member in a serodiscordant partnership who will be interviewed alone*

*Objectives*:

1. Describe the HIV uninfected members’ experiences with PrEP delivery including facilitators and impediments to access to PrEP services integrated in HIV clinics
2. Identify factors that influence individual’s decision to initiate PrEP and continue PrEP including understanding of and confidence in PrEP effectiveness, health providers attitudes, and accessibility of (?) the heath system.
3. Explore the HIV uninfected members’ views and confidence in treatment as prevention (U=U)

With these interviews among individual HIV uninfected partners, we are trying to capture information relevant to service delivery at specific clinics, as well as the individual’s perceptions and experience with ARV-based HIV prevention.

# In depth Interview guide: HIV- Individuals

## **INTRODUCTION**

Welcome to our discussion. Please feel free to share anything you want with me.

## **Topic 1: DECISION TO START PrEP**

- Before you started using PrEP, what did you know about PrEP?
- How did you learn about PrEP?
- Why did you decide to start taking PrEP?
  - Was it your decision to start PrEP, or was it the health provider’s?
  - If provider, how confident were you in what the provider told you about PrEP?

## **TOPIC 2: PREP ACCESS/DELIVERY PROCESS**

- Tell me about the process you go through to get PrEP in this clinic? What happens to you when you walk into this clinic to get PrEP? What are some of the concerns or fears you heard about getting PrEP from this clinic? (Probe for provider attitude, provider knowledge, waiting time-are they spending more/less time, delivery process)
- What makes it challenging for you to receive services in this clinic? (Barriers)
- What has made it easier for you to receive PrEP services in this clinic? (Facilitators)
- What do you forego to come for PrEP services? (opportunity cost)

## **Topic 3: HIV UNINFECTED PERSON’S EXPERIENCES OF SEEKING SERVICES IN A HIV CLINIC (STIGMA)**

- How confident or comfortable are you that you can get PrEP from a hospital/clinic anytime you need it (on scale of 1-10) why?
- How do you feel about accessing PrEP services from this clinic?
- One of the things facilities do is that, when one is tested for HIV and found to be HIV infected, the health care provider follows them at home to test their partner(s) and link them up for PrEP if they are negative or for ARV for treatment.
  - How do you feel about this approach?
  - What do you think are the benefits or challenges for this?
- What do people say when they see you getting services from a HIV clinic? (if not yet heard probe “What would people say?)
- Where would you prefer to access PrEP services from? Why? (Location, another facility, another delivery point)
  - What would make it easier for you to receive PrEP?

## **Topic 4: COUPLENESS**

- How often do you come with your partner to the clinic? Why/Why not?
- What has been your experience at the clinic when you come with your partner (as a couple) compared to when you come as an individual?
- How has taking PrEP impacted your relationship? Or has your relationships changed since you started taking PrEP? If so, how?
- How has your HIV positive partner benefited from you taking PrEP?

## **Topic 5: ADHERENCE AND RETENTION**

**Adherence:**

What is it like to take PrEP every day?

- How do you take PrEP?
- What do you like about taking PrEP?
- What do you dislike about taking PrEP?
- What helps you remember to take PrEP?
- What challenges have you faced since you started PrEP?
- Before you started PrEP, what were your concerns about taking it?
- Have any of the things you were concerned about actually been challenges for you? Have you developed any solutions? What are they?

Are there times when you stopped taking PrEP?

- Why, duration?
- How did you feel?
- How did that influence whether/how you had sex?

**Retention:**

- How often do you come to this clinic for your PrEP? What would you prefer?
- Does having your HIV infected partner in this clinic influence your retention? How?
- How well do you keep the appointments? If you have ever missed an appointment what were the reasons?
- What helps you keep your clinic appointment?
- What could be done to make it easier for you to continue being on PrEP?

## **Topic 6: COMMUNITY PERCEPTIONS**

- What are people saying in the community about PrEP? (If people in your community hear that you are taking PrEP, what would they say?)-Probe: Who they think should be on PrEP?
  - How has this influenced your coming to the clinic?
  - How has it influenced how you take your drugs as advised by your doctor?
- Who are the people who know that you are taking PrEP?
  - What did they tell you or do when they learnt that you are taking PrEP?
  - How has this influenced the way you take PrEP or come back for your appointment?
- Do you know anybody else taking PrEP in your community?
  - Why are they taking PrEP?
  - How has this influenced your taking of PrEP?

## **Topic 7: COUNSELLING**

- Tell me what the healthcare providers told you about PrEP?
  - What did they tell you how well PrEP works to prevent HIV infection?
  - What did they tell you about how long you will take PrEP or when you can stop it?
  - What did they tell you about side effects?
  - What are some of the questions or concerns you had about what you were told?
  - How well do you think PrEP works to prevent HIV infection?

**Condoms**

- What are your thoughts about use of condoms with PrEP?
- What have health providers told you about condom use with PrEP?
  - What are some of the questions you had/have about what you were told?
- Tell me about your experiences with condoms before you started PrEP? Has this changed since you started taking PrEP? How?

**Viral suppression U=U (For interviewees with a HIV infected partner)**

- When do you think is the right time for you to discontinue PrEP?
- What have health providers told you about how well treating the HIV infected partners with ART works to reduce HIV transmission?
- (If client doesn’t know read: Treating HIV infected people with ART reduces their viral load and the chances of them passing the HIV to HIV-uninfected partners)
  - How confident are you that when your partner’s HIV viral load is undetectable after treatment that it will prevent you from getting HIV? How confident were you in what the provider told you?
  - What are some of the questions you had about what you were told?
- Why would some people continue taking PrEP even after their partners have undetectable viral load?
- If you learnt today that your partner has undetectable viral load, would you stop taking PrEP? Why/why not?
