# Supplementary material for: “I just believe there is a risk” understanding of undetectable equals untransmissible (U = U) among health providers and HIV‐negative partners in serodiscordant relationships in Kenya
Source: J Int AIDS Soc. 2020 Mar 6;23(3):e25466. doi: 10.1002/jia2.25466 (PMC7060133; doi:10.1002/jia2.25466)
Supplement: Supplementary file 3 [file JIA2-23-e25466-s003.docx]

**Data S1**:- Health provider interview guide.

**Data S2**:- PrEP user interview guide.
